# Clostridial Neurotoxins: Structure, Function and Implications to Other Bacterial Toxins

**DOI:** 10.3390/microorganisms9112206

**Published:** 2021-10-23

**Authors:** Shuowei Cai, Raj Kumar, Bal Ram Singh

**Affiliations:** 1 Department of Chemistry and Biochemistry, University of Massachusetts Dartmouth, Dartmouth, MA 02747, USA; 2 Botulinum Research Center, Institute of Advanced Sciences, Dartmouth, MA 02747, USA; rkumar@inads.org (R.K.); bsingh@inads.org (B.R.S.)

**Keywords:** Gram-positive bacterial toxins, clostridial neurotoxin, botulinum neurotoxin, tetanus neurotoxin, molten globule, diphtheria toxin, anthrax toxin, pore-forming toxins, proteolytic activation

## Abstract

Gram-positive bacteria are ancient organisms. Many bacteria, including Gram-positive bacteria, produce toxins to manipulate the host, leading to various diseases. While the targets of Gram-positive bacterial toxins are diverse, many of those toxins use a similar mechanism to invade host cells and exert their functions. Clostridial neurotoxins produced by *Clostridial tetani* and *Clostridial botulinum* provide a classical example to illustrate the structure–function relationship of bacterial toxins. Here, we critically review the recent progress of the structure–function relationship of clostridial neurotoxins, including the diversity of the clostridial neurotoxins, the mode of actions, and the flexible structures required for the activation of toxins. The mechanism clostridial neurotoxins use for triggering their activity is shared with many other Gram-positive bacterial toxins, especially molten globule-type structures. This review also summarizes the implications of the molten globule-type flexible structures to other Gram-positive bacterial toxins. Understanding these highly dynamic flexible structures in solution and their role in the function of bacterial toxins not only fills in the missing link of the high-resolution structures from X-ray crystallography but also provides vital information for better designing antidotes against those toxins.

## 1. Introduction

**Figure 1:**
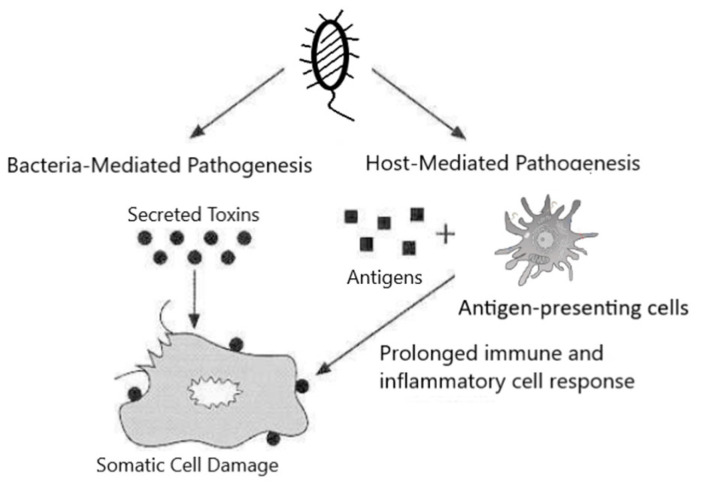
Mechanisms of bacterial pathogenesis: bacteria-induced toxicity and host-mediated damage [2].

The bacterial toxin, produced by bacteria as a virulent factor, is one of the main mechanisms bacteria use to manipulate the host, leading to various diseases, some even fatal. Bacterial toxins can be classified as exotoxins and endotoxins. The former are synthesized inside bacteria and secreted (some exotoxins are only released by lysis of the bacterial cell), while the latter are part of the bacterial membrane and are only released after bacterial cells are killed. Based on their chemical compositions, bacterial toxins can be divide into two types: lipopolysaccharides, which are associated with bacterial cell walls and released after disruption of the cell (endotoxins), and protein (or, minimally, peptides) toxins, which are synthesized inside the cells and then released (exotoxins) [1]. Endotoxins trigger a series of host cell responses following exposure to the host, and that plays a major role in the tissue damage and pathogenesis of endotoxins (host-mediated pathogenesis). Contrary to the extensive systemic and immune responses to the endotoxin in the host, the site of action of most exotoxins is more localized and is confined to particular cell types or cell receptors (bacteria-mediated pathogenesis) (Figure 1) [2].

While lipopolysaccharide endotoxin is a structural component of all Gram-negative bacteria, exotoxins are produced by both Gram-positive and Gram-negative bacteria and form a class of poisons that is among the most potent, per unit weight, of all toxic substances [2]. For many bacterial protein toxins, the toxins themselves are the pathogenic factor and do not necessarily involve bacterial infections (toxinosis) [3]. They act either enzymatically or through direct action toward host cells and lead to a variety of host responses [4].

Based on their modes of action, bacterial protein toxins can be classified into three types [5]: type I toxins mediate the host response and disrupt the host without entering the cells, such as superantigens produced by *Staphylococcus aureus* and *Streptococcus pyogenes*. Type II toxins damage host cell membranes to invade and disturb host defense processes within the cell (pore-forming toxins). Some examples of type II toxins include exotoxins of *Clostridium perfringens* and exotoxins of *Clostridium difficile*. Type III toxins are often called A–B toxins due to their binary structure. They usually act at tissues sites remote from that of bacterial invasion or growth. The B component of these toxins serves as the receptor-binding domain that binds to the receptor on the host cell surface, while A component possesses the enzymatic activity to damage the cell. Examples of this group of toxins include diphtheria toxin, anthrax toxin, and clostridial neurotoxins.

Based on their biological effect on host cells, exotoxins also can be grouped as neurotoxins, cytotoxins, and enterotoxins [2,6]. Clostridial neurotoxins produced by *Clostridium* spp., including botulinum neurotoxins and tetanus toxin, are among the best examples of neurotoxins [7,8]. Cytotoxins are a large group of toxins with a wide range of host cell specificities and toxic actions. For example, diphtheria toxin, which is produced by Gram-positive Corynebacterium diphtheriae, inhibits protein synthesis in many cell types by catalyzing the ADP-ribosylation of elongation factor II and blocking the elongation of the growing peptide chain [9]. Enterotoxins increase the hypersecretion of water and electrolytes from the intestinal epithelium by activation of the membrane pores through increased cAMP or by an increased calcium ion concentration intracellularly. The pore formation changes the osmolarity of the luminal contents of the intestines. An increased chloride permeability leads to leakage into the lumen followed by sodium and water movement, causing secretory diarrhea [10]. They are cytotoxic and kill cells by forming pores and increasing the apical membrane permeability of the mucosal cells of the intestinal wall [11]. Several Gram-positive bacteria secrete enterotoxins and lead to food poisoning, including *Staphylococcus aureus* and *Bacillus cereus*, causing *Staphylococcal* food poisoning and *Bacillus cereus* diarrheal disease, respectively [11].

Like all proteins, the structure of bacterial protein toxins governs their biological functions. For many protein toxins produced by Gram-positive bacteria (and Gram-negative), their structures need to be activated to exert their biological activities to the host cells. The activation can be either through the proteases produced by those bacteria or the host cells’ local environments. Clostridial neurotoxins provide a classical example for understanding the mechanisms of bacterial toxins, particularly for A–B-type toxins. Here, we will critically review the structure–function aspect of clostridial neurotoxins and the relationship between the flexible structures of protein toxins and their biological activities. Many Gram-positive bacterial toxins use a similar approach to be activated, and flexible structures are also required for their biological functions. Therefore, we will also discuss the implications of other Gram-positive bacterial protein toxins.

## 2. Clostridia Neurotoxins

Clostridial neurotoxins include botulinum neurotoxins (BoNTs) and tetanus neurotoxin (TeNT). They are the most potent toxins to mankind. Botulism and tetanus are responsible for severe neurological diseases, respectively. While BoNTs cause flaccid paralysis, TeNT causes spastic paralysis [7,8]. Eventually, both BoNTs and TeNT will lead to respiratory failure and death.

Tetanus is mainly caused by deep penetrating wounds where anaerobic bacterial growth is facilitated [12]. Human botulism, however, can occur in one of three natural forms: foodborne, wound, and intestinal (infant botulism and adult sporadic botulism) [13,14]. While inhalational botulism does not occur naturally, it is a concern as a potential bioterror attack [15]. With the increased use of BoNT (especially BoNT/A) as therapeutic agents for neuromuscular disorders and medical aesthetics, iatrogenic botulism is also of concern [16]. Foodborne botulism is caused by ingestion of the preformed toxin from contaminated food. Wound botulism is rare (but increasing among injecting drug abusers) and results from the growth of *C. botulinum* spores in a contaminated wound with in situ productions of toxin. Intestinal botulism is caused by colonization (infection) of the intestine by spores of *C. botulinum*, with subsequent in situ toxin production. Intestinal botulism is often infectious to infants, as the gut microflora of small babies is poorly developed, but can also infect adults with previous bowel surgery, bowel anomalies, or the recent use of antimicrobials that may disrupt the normal intestinal flora [17]. While medical treatment with BoNT has a very good safety profile and tolerability, an extremely rare but severe complication of BoNT therapy presenting like botulism can occur and is known as iatrogenic botulism [16,18,19]. Foodborne and intestinal botulism (infant) are the most common forms of human botulism, and both require an uptake of BoNT through the gastrointestinal tract, a highly unusual phenomenon for a large protein like BoNT.

### 2.1. Diversity of Clostridial Neurotoxins

#### 2.1.1. Tetanus Neurotoxin

TeNT is produced by *Clostridium tetani*, while BoNTs are produced by *Clostridium botulinum*. Both *C. tetani* and *C. botulinum* are strictly anaerobic Gram-positive bacteria. Forty-three *C. tetani* strains have been isolated [20], most from human wounds, and their whole-genome sequences have been reported [21]. Strains of *C. tetani* are classified into two main groups: the Harvard strains (derived from the ancestral Harvard strain) and the wild-type strains isolated from clinical cases. Strain-specific proteins are mainly found in their prophage regions and their CRISPR/Cas loci and spacer regions [22]. The toxin-encoding genes are highly identical; thus, there is only one type of TeNT [20,22].

#### 2.1.2. Botulinum Neurotoxin

Both *C. botulinum* strains and the BoNTs that they produce are very heterogeneous compared to *C. tetani* strains and the tetanus neurotoxin. Based on the neurotoxin neutralization assay using serotype-specific antisera, seven serotypes of BoNTs have been confirmed (type A–G) [1,7,23,24]. A potential new serotype, type H, was found in 2013 [25], which was later identified as a hybrid between type A and type F and also called BoNT/FA or BoNT/HA [25,26,27,28,29,30]. Furthermore, another new serotype of BoNT, BoNT/X, was identified recently with a low sequence identity with other serotypes of BoNT and is not recognized by antisera against BoNT/A-/G [31,32].

The diversity of BoNTs is not just reflected in that they include several serotypes. Based on the amino acid sequence variations, each serotype of BoNT is also divided into subtypes. A numerical notation is added to each serotype to designate those subtypes, i.e., BoNT/A1, BoNT/A2, BoNT/A3, etc. [33]. With the development of DNA sequencing, an increased number of the individual genes of *C. botulinum* have been sequenced, and more subtypes have been identified. Currently, there are eight subtypes of BoNT/A (A1-A8), eight subtypes of BoNT/B (B1-B8), two subtypes of BoNT/C (C1 and CD, a chimera composed of a 2/3 type C and 1/3 type D sequence), two subtypes of BoNT/D (D and DC, a mosaic toxin with a 2/3 type D and 1/3 type C sequence), twelve subtypes of BoNT/E (E1-E12), and eight subtypes of BoNT/F (F1-F8) [33]. A subtype can affect the antibody binding and neutralization and the catalytic efficiency for their respective substrate [34,35,36,37,38,39].

Heterogeneous *C. botulinum* groups into four discrete groups (*C. botulinum* Groups I-IV) with the common feature of forming botulinum neurotoxin [26]. Group I of C. botulinum (proteolytic C. botulinum) is a highly proteolytic and mesophilic bacterium forming very heat-resistant spores [40]. Group I of C. botulinum is a major cause of human botulism (foodborne, infant, and wound), and strains of Group I *C botulinum* produce one or more neurotoxins of types A, B, or F [41]. The number of toxin genes in the Group I strains and the number of toxins produced are variable, and it can process up to three neurotoxin genes and produce one or more different serotypes of neurotoxins [33,42]. BoNT/X was identified from Group I *C. botulinum* strain 111, a strain originally isolated from an infant botulism case in Japan as the first case of type B infant botulism, where BoNT/B2 (encoded on a plasmid) was identified as the neurotoxin agent [43,44,45]. The cultures and colonies of strain 111 lost toxicity after 10 or more passages, suggesting the loss of the neurotoxigenic plasmid [46]. However, based on the sequence of strain 111 deposited at GeneBank, a recombinant version of this toxin has been produced, and the toxin cannot be recognized by the existing antisera and, therefore, is designed as BoNT type X (BoNT/X) [31,32]. *C. botulinum* Group II (nonproteolytic *C. botulinum*) is a saccharolytic and psychrotrophic bacterium that forms spores of moderate heat resistance [40,47]. The strains of Group II produce a single neurotoxin of type B (B4), E, or F (F6) and is an important cause of foodborne botulism. While not known to produce multiple toxins, the sequencing of the genomes of the Group II BoNT/F6 strains also shows the fragments of a type B and a type E neurotoxin gene [48]. The strains of *C. botulinum* Group III produce a single neurotoxin of type C or type D or, more often, a hybrid neurotoxin compromising elements of C and D (type C/D or D/C). Group III *C. botulinum* is saccharolytic and mesophilic bacterium forming spores of high heat [40]. The strains of Group III *C. botulinum* are responsible for botulism in a wide range of animal species [49,50,51]. *C. botulinum* Group IV (also known as *Clostridium argentinense*) strains produce BoNT/G, which has not been definitively associated with human or animal botulism [52]. In addition to *C. botulinum*, some strains of *Clostridium baratii* can produce BoNT type F (F7), and some strains of *Clostridium butyricum* can produce BoNT/E (E4 or E5); both can cause human botulism [53]. Accordingly, two additional groups have been included in heterogeneous *C. botulinum*: Group V encompasses BoNT/F-producing *C. baratii* strains, and Group VI encompasses BoNT/E-producing *C. butyricum* strains [54].

Toxin-related gene clusters in the botulinum family are moderately conserved with about 40% identity but located in diverse locations in the genome (either on the chromosome, plasmid, or bacteriophage loci, depending on the *C. botulinum* stains) [22,55]. *C. tetani*, on the other hand, is simpler, carrying a single plasmid encoded only with TetX (TeNT gene) and TetR (TetX transcriptional regulator gene) [22].

BoNT genes are also found in non-Clostridial organisms. By using bioinformatics tools, BoNT gene clusters are found in *Weissella oryzae* SG25 (Wo-ORF1) [56], *Enterococcus* sp. 3G1_DIV0629 [57], and *Chryseobacterium piperi* [58]. Wo-ORF1 was produced through recombinant methods and showed a cleavage of VAMP-2 (the substrate of BoNT/B) [59]. This BoNT homolog was called BoNT/Wo. The new homolog putative neurotoxin identified from *Enterococcus* sp. 3G1_DIV0629 was initially called eboNT/J [57], and later, the toxin was produced using recombinant methods and was called BoNT/En [60]. BoNT/En cleaved VAMP-1, -2, and -3 at a conserved peptide bond, A67-D68, and also cleaved syntaxin-1B, syntaxin-4, SNAP 23/25 [60], and all SNARE proteins targeted by BoNTs but did not show a toxicity in mice. When BoNT/En-LC merges with heavy-chain BoNT/A, it leads to paralysis in mice, suggesting that mice neurons maybe lack a receptor for BoNT/En [60]. This high diversity of BoNTs may be the result of horizontal gene transfer [1,61].

Remarkably, BoNTs are not just the most toxic substance to mankind, they are also an effective medicine for a wide range of neuromuscular disorders and are used for medical aesthetics [62,63]. The diversity of BoNTs is not only important for bacteriology and infectious diseases but also provides a pool of candidates for the new therapeutical applications of BoNTs.

### 2.2. Mode of Action of Clostridial Neurotoxins

**Figure 2:**
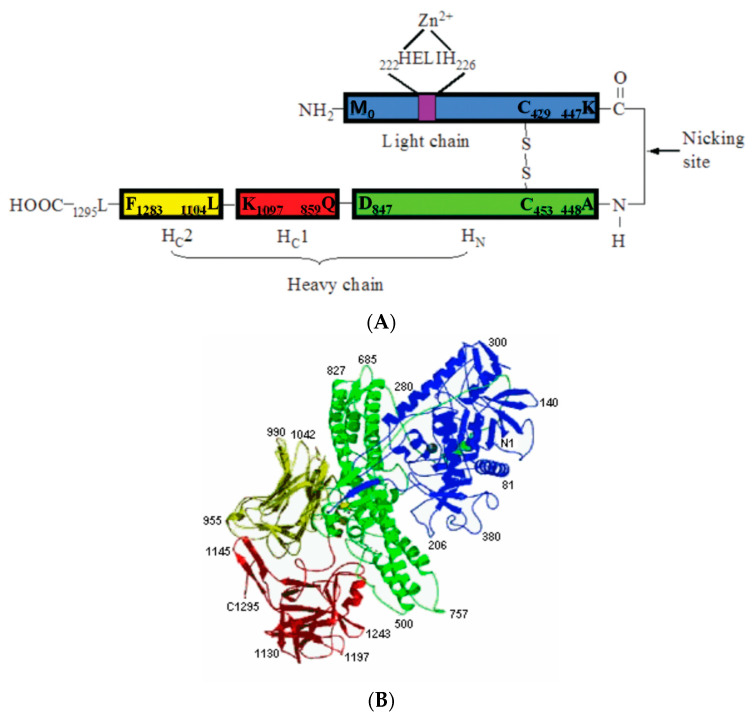
(**A**) Schematic diagram of a BoNT (BoNT/A) showing different domains. (**B**) Three-dimensional representation of the backbone BoNT/A [66]. The catalytic domain LC is indicated in blue, the translocation domain HN is in green, and the receptor-binding domain HCC and HCN are in yellow and red, respectively.

Clostridial neurotoxins are typical A–B-type toxins. Both TeNT and BoNT are synthesized as a single-chain precursor protein of about 150 kDa that is inactive or just weakly active (Figure 2) [8]. The single-chain precursor protein does not have a signal peptide and is released from bacteria through cell wall exfoliation [64,65]. The single-chain precursor protein is proteolytically activated in the extrabacterial medium either by *Clostridium* proteases or by exogenous proteases (such as digestive proteases in the intestine) into a dichain form linked through an interchain disulfide bond (Figure 2). The light chain (LC) is about 50 kDa, while the heavy chain (HC) is about 100 kDa. The heavy chain is responsible for the recognition of the presynaptic nerve terminals, binding to nerve cells, and transferring the LC into the cytosol through receptor-mediated endocytosis. The LC behaves as endopeptidase specifically targeted at one of the three members of the soluble N-ethylmaleimide-sensitive factor attachment protein receptor (SNARE) proteins and inhibits neurotransmitter release.

#### 2.2.1. Uptake of Botulinum Neurotoxins in the GI Tract

##### Progenitor Toxin Complexes of BoNT

BoNTs are produced by toxigenic strains of *Clostridium* together with several associated nontoxic proteins, while TeNT is produced by *C. tetani* without any toxin-bound accessory proteins. BoNTs and TeNT have different infectious routes; the majority of botulism cases in adults are foodborne botulism caused by the ingestion of BoNTs, while TeNT is mainly infected by wounds. The nontoxic-associated proteins of BoNTs have long been thought to help BoNTs absorb into the GI tract [67]. Without the accessory proteins, however, TeNT is not toxic via ingestion.

The genes encoding nontoxic-associated proteins are arranged in a cluster adjacent to the neurotoxin gene (bont gene) [68]. A large nontoxic nonhemagglutinin protein of 139 kDa coded by a gene ntnh upstream to the bont gene is present in all seven serotypes of BoNT and is called the neurotoxin-binding protein (NBP, also called NTNHA) [69,70,71]. NBP is coexpressed with neurotoxin. In addition to ntnh, neurotoxin gene clusters also include ha (producing hemagglutinin proteins HA70, HA17, and HA33) and orf-X gene clusters [68]. The orf-X gene clusters include three open reading frames (orfX1, orfX2, and orfX3) that have been shown to produce proteins that form somewhat fragile toxin complexes [56,72,73]. Collectively, BoNTs are produced as neurotoxin complexes (progenitor toxin complex or PTC) of different sizes. The botR gene, a transcriptional regulator, and p47 are also located within the bont gene clusters. The P47 protein seems to share a structural domain with the orfX2 and orfX3 proteins, and that is related to the TULIP family of the lipid-binding proteins [74,75,76].

The NBP–BoNT heterodimer is present for all seven serotypes of BoNTs. The NBP–BoNT complex may or may not progress to the formation of larger complexes associated with neurotoxin-associated proteins (NAPs). Three forms of PTCs have been characterized: extra-large size (LL complex, sediments at 19S, about 900 kDa), large size (L complex, sediments at 16 S, about 500 kDa), and medium size (M complex, sediments at 12 S, about 300 kDa). The M complex is the BoNT–NBP complex, and the L complex is composed of the BoNT–NBP complex and several neurotoxin-associated proteins (NAPs) with hemagglutinin (HA) activity, while the LL complex is believed to result from the association of two L complexes [69]. Depending on the strain, the bacterium produces one, two, or all three forms of PTCs [77]. BoNT/A1 is produced by *C. botulinum* in all three forms of the complex; BoNT/B, /C, and /D are produced in two forms: the 16 S L complex and 12 S M complex. BoNT/G is produced as the 16 S L complex. BoNT/E and /F are only produced as the M complex [78]. While strains that produce BoNT/E and /F lack ha gene clusters, they have orfX clusters, with orfX1, orfX2, and orfX3, along with p47 [79]. OrfX2 has been identified in type E, but not in type F, and orfX2 and P47 are in both serotypes [80,81]. Orf BoNT complexes are unstable [72,73,82], but the BoNT/E L complex with the P70 protein (a 70-kDa NAP) and P18 protein (an 18-kDa NAP) has been purified from the bacterial culture [83].

##### Protection of BoNT in GI Tract by Nontoxic Proteins

The NAPs have long been thought to be associated with the oral toxicity of BoNTs [63]. Hemagglutinin 33 (Hn-33, also called HA-33) from BoNT/A was the first hemagglutinin protein purified and characterized from the BoNTs [84]. Hn-33 showed a strong structural stability at a low pH and was highly resistant to proteases, including GI tract proteases (pepsin and trypsin) [85]. More importantly, when complexed with the pure toxin, Hn-33 showed a protection of BoNT/A from proteolysis [85]. In addition to HA proteins, NBP could also provide a protection of BoNT in the GI tract. Based on the X-ray crystal structure of 12 S PTC of BoNT/A1, NBP has a similar structure to BoNT and, in the complex form, provides large and multivalent binding interfaces to shield the C-terminal domain of the heavy chain of BoNT [86]. Evidently, TeNT forms no complex, and without those nontoxic proteins, TeNT is not active through the oral route.

##### Uptake of BoNT through the Intestinal Epithelial Barrier

**Figure 3:**
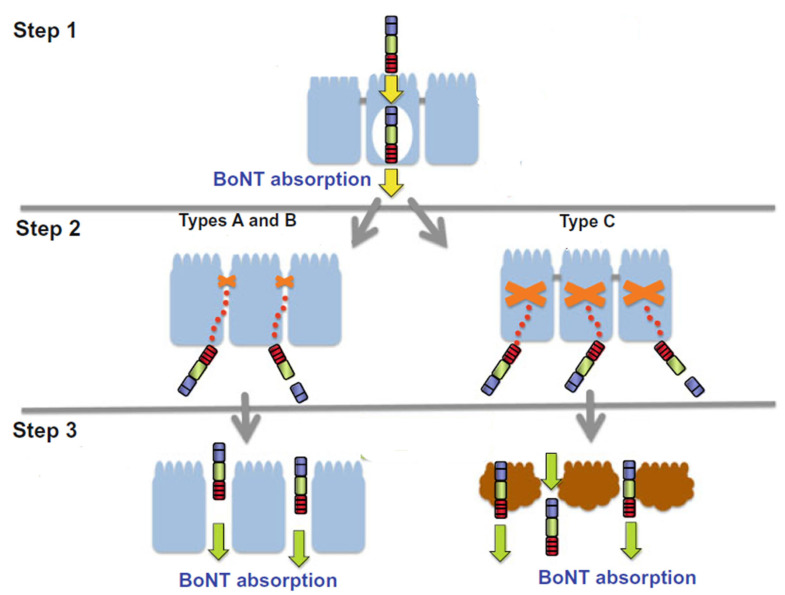
The proposed model for BoNT across the intestinal epithelial barrier (modified from Fujinaga et al., 2012 [77]). Step 1: NAPs/hemagglutinin proteins facilitate the toxin transcytosis through the epithelial cells. Step 2: HA and/or other NAPs mediate the disruption of the epithelial barrier (BoNT/A and /B disrupt the tight junction, while BoNT/C is cytotoxic to the epithelial cells. Step 3: BoNT is absorbed from the disrupted cellular barriers.

The intestine surface is lined with a continuous monolayer of epithelial cells to restrict the passage of harmful molecules from the luminal surface. Once reaching the intestine, in addition to proteases, BoNT must pass through the lumen surface of the small intestine to reach the systematic circulation. A three-step hypothesis has been proposed for BoNT across the intestinal epithelial barrier (Figure 3) [77].

Step 1. a small amount of toxin undergoes transcytosis across the epithelial cells without disrupting the tight junction of the epithelial barrier. Both pure toxin and the BoNT complex can undergo transcytosis through epithelial cells but potentially through different mechanisms. It has been shown that BoNT alone can go through transcytosis of the epithelial monolayer through a receptor-mediated mechanism [87,88,89,90,91,92]. The heavy chain of BoNT/A and its C-terminus fragment (HC) have shown binding to the internal cell lines, and the ganglioside (GD1b and GT1b) and SV2C proteins (or related proteins) expressed on these cells involve transcytosis [90]. Further, the binding of BoNT/A to a mouse crypt-like cell line (m-ICc12) with higher levels of SV2C is greater than Caco-2 and has a 10-fold higher transcytosis rate than Caco-2 [91]. The receptor-mediated transcytosis of epithelial cells is only examined in BoNT/A.

Evidence also shows that HA proteins of BoNT are pivotal for the transcytosis of BoNT through the epithelial monolayer. The carbohydrate-binding activity of HA may facilitate the adhesion of toxins to the intestine cells. Fujinaga et al. first reported that the type C 16S complex (but neither the 12S complex or the toxin alone) binds to the carbohydrates expressed on Guinea pig intestinal epithelium [93]. Recently, Ghosal et al. showed that the BoNT/A complex was internalized into the human intestinal epithelial cell (HT-29) monolayer faster than pure BoNT/A, suggesting that NAPs play a critical role and facilitate the transcytosis of toxins across the intestinal barrier [94]. In addition to intestinal epithelial cells, Hn-33 also facilitated the transcytosis of toxins across human bronchial epithelial (HBE) cells, suggesting its roles in inhalational botulism [94]. HAs from other serotypes are also shown to bind to intestinal epithelial cells [77]. For BoNTs’ lack of HAs, other NAPs may play roles in the transcytosis of BoNTs at the intestinal epithelium level. NAP-70, a 70-kDa NAP from BoNT/E without HA activity, showed the transcytosis of BoNT through Caco-2 cell monolayers and disrupted the tight junctions [95].

Step 2. Once BoNT is on the basolateral surface, the HA and/or other NAPs mediate the disruption of the epithelial barrier. It has demonstrated that type B HA disrupts the Caco-2 cell and MDCK I (canine kidney epithelial) cell monolayers by acting on the basolateral side and disrupts the tight and adherens junction proteins (occludin, ZO-1, β-catenin, and E-cadherin) at the cell-to-cell boundaries without affecting the viability of the cells [96]. BoNT/A and /B HA proteins exhibit a similar mechanism to disrupt the epithelial cell monolayers with different cell lines (Caco-2, T84 (a human colon-originated epithelial cell line), and MDCK I) [97]. It has been shown that epithelial cadherin (E-cadherin) is the target for HA proteins from BoNT/A and B. HAs from BoNT/A and /B bind E-cadherin directly and disrupt the intercellular barrier without causing cytotoxic effects in epithelial cells of susceptible hosts [98]. HA from BoNT/C, on the other hand, showed a different mechanism through cytotoxicity when using rat colon carcinoma cell lines (ACL-15 and RCN-9) [97]. The ability of interaction of human E-cadherin by the BoNT/A and /B complexes, but not the BoNT/C complex, may help explain that BoNT/A and /B are more common causes of human botulism than BoNT/C.

Step 3. The disrupted cellular barrier (either through the disruption of tight junctions by BoNTA and /B or through cytotoxicity by BoNT/C) allows the paracellular passage of a large quantity of the BoNT complex or BoNT alone [77].

#### 2.2.2. Molecular Mechanisms of Clostridial Neurotoxins toward the Targeted Neuronal Cells

Once being absorbed and crossing the intestine barrier, BoNTs are delivered to the extracellular space, and some are in the general circulation (blood and/or lymph) [99,100,101]. While only a small amount of BoNTs reach their targeted neuron cells and cause the symptoms of botulism, the exact route of the dissemination of BoNTs to their targets is still not well-understood. Locally, BoNTs interact with the enteric nervous system (ENS), but not all neurons are recognized by BoNTs [92,102,103]. The dissemination of BoNTs in the body through blood and lymph circulation allows BoNT molecules to reach the peripheral nerve endings [102,104,105]. However, there is also another hypothesis that BoNTs are disseminated by axonal transport, as is the case for TeNT. This hypothesis is based on the fact that botulism symptoms are characterized as descendent flaccid paralysis starting with an attack on the cranial nerves. If the bloodstream is the main route for BoNT dissemination, it could distribute to the entire neuromuscular junction, leading to generalized botulism symptoms [99]. In fact, it has been shown that BoNT/A undergoes retrograde transport to the motor neurons and central nervous system and is then transcytosed to the afferent neurons [106].

Unlike BoNTs that mainly target the motor neuron endings at the neuromuscular junctions, TeNT only uses motor neurons and, through a transcytototic mechanism, targets the neurons in the central nervous system (see the retrograde transport of TeNT section below).

**Figure 4:**
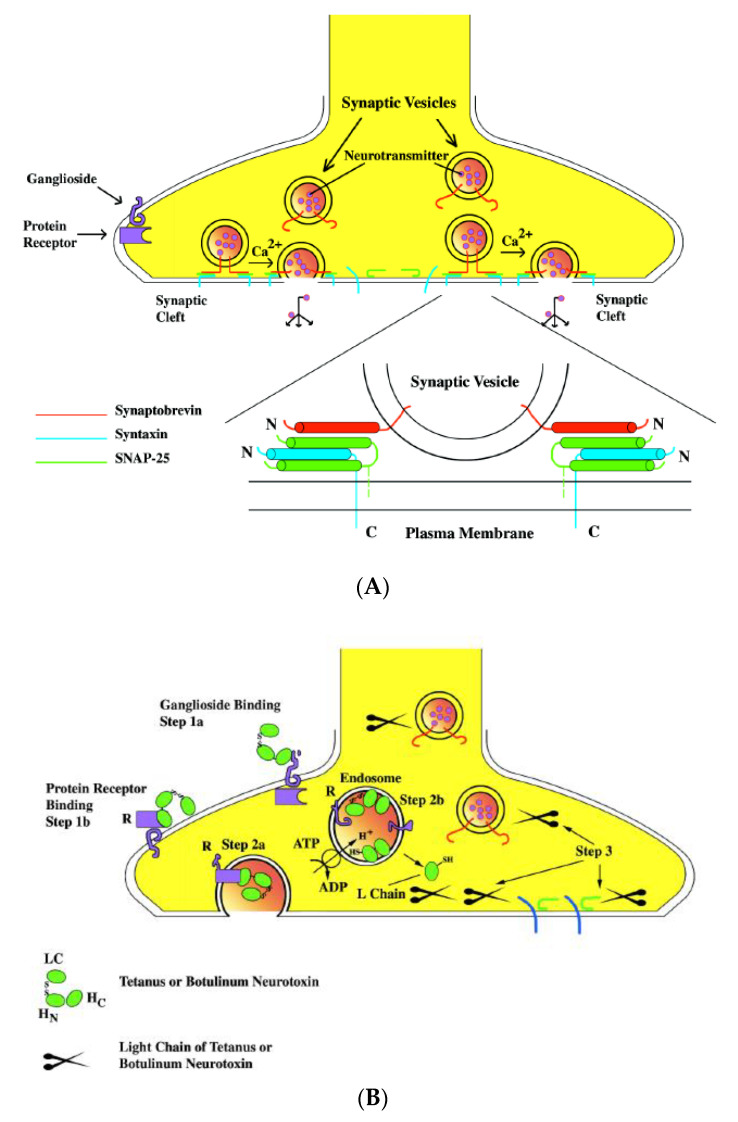
Schematic models of the neurotransmitter release and the actions of botulinum and tetanus toxins [108]. (**A**) Synaptic vesicles containing neurotransmitters dock with the plasma membrane through SNARE proteins (synaptobrevin, syntaxin, and SNAP-25). SNARE proteins remain in random coil conformations until associated with the SNARE complex at docking, where they form a helical bundle. (**B**) Botulinum or tetanus toxin binds to the presynaptic membrane through gangliosides and a protein receptor (step 1) internalized through endocytosis (step 2a), and its LC is translocated across the membrane (step 2b). The LC acts as a specific endopeptidase against either synaptobrevin (on synaptic vesicles), syntaxin (on the plasma membrane), or SNAP-25 (on the plasma membrane) (step 3). BoNTs (or TeNT) cleave their substrates before the SNARE complex is formed.

When targeting neurons, BoNTs and TeNT share a similar mechanism. In A–B-type toxins, the heavy chains of BoNTs and TeNT serve as the receptor-binding domain, while the light chains serve as the enzymatic domain intracellularly (Figure 2 and Figure 4). A three-step intoxication mechanism was proposed more than two decades ago [107,108]: binding and receptor-mediated endocytosis, translocation, and intracellular enzymatic activity targeting SNARE proteins (Figure 4).

##### Binding to the Neural Cells

The first step of the action of the clostridial neurotoxin is binding to the neural cells. The C-terminal domain of HC (H_C)_ is believed to be the receptor-binding domain. From the X-ray crystal structure, H_C_ is further divided into two distinct folds, a carboxyl-terminal b-trefoil (H_CC_) with an amino-terminal lectin-like jelly roll (H_CN_) [66,109,110,111,112]. HCC is highly diverse in the sequences among TeNT and different serotypes of BoNT, while HCN is highly conserved. A double-receptors model was proposed by Montecucco [113]. Clostridial neurotoxins first bind to the ganglioside of the neural membrane, preferentially to the b-series. The G1b series of gangliosides (GT1b and GD1b), as well as GD1a, have shown the ability to bind to both TeNT and BoNTs [114]. The ganglioside-binding sites are located on the C-terminal half of the receptor-binding domain (H_CC_). Through the cocrystallization of toxin and ganglioside, a conserved core residues SxWY….G of a WY loop at HCC has been identified as the ganglioside-binding site [115,116]. The SxWY core residues are conserved among TeNT, BoNT/A, /B, /E, /F, and /G [117]. SxWY is not conserved in BoNT/C and /D, but mutagenesis studies and cocrystallization suggest that their positions in BoNT/C and /D are analogous to the conserved WY loop in other BoNTs as a ganglioside-binding pocket and contribute to ganglioside binding [118,119]. From cocrystallization and mutagenesis studies, a second ganglioside-binding site (a sialic acid-binding site) has also been identified for TeNT, BoNT/C, and BoNT/D [116,117,118,119].

The binding between ganglioside and clostridial neurotoxins is weak. However, the widely distributed gangliosides may be responsible for accumulating clostridial neurotoxins on neuronal surfaces and lead to more specific and stronger bindings to protein receptors. Synaptotagmin (SYT) and synaptic vesicle glycoprotein 2 (SV2) have been identified as the protein receptors for BoNTs (Table 1). The protein receptor for BoNT/C has not been identified, and it may use unknown SV structures as the secondary receptor [118,120,121]. The cocrystal structure of the toxin and protein receptor revealed that the protein receptor also binds to the H_CC_. Through a ternary crystal structure of the HC/B-SYT–ganglioside complex, the structure demonstrated that the ganglioside-binding site and SYT-binding site are isolated from each other, suggesting that the ganglioside-binding site and protein receptor-binding site are two independent anchor sites in the double-receptor hypothesis [111]. This raises the question of the function of the H_CN_ motif. H_CN_ has shown binding to sphingomyelin-enriched membrane microdomains and can serve a function in priming the translocation domain to become an insertion competent to the orientation of the membrane [122].

##### Internalization and Translocation

The targets of clostridial neurotoxins are their cytosolic SNARE proteins; therefore, the neurotoxin or, at least, its catalytic domain has to reach the cytosol of the nerve cells to block neurotransmitter exocytosis. Clostridial neurotoxins do not enter the cell directly from the plasma membrane; rather, they are endocytosed inside acidic cellular compartments [107]. Electron microscopic investigations show that, after binding, clostridial neurotoxins enter the lumen of vesicular structures in a temperature and energy-dependent process [137,138,139]. The receptor-binding domain H_C_ of clostridial neurotoxins appears to be sufficient for the internalization process in murine spinal cord neurons [140].

Following the internalization, the clostridial neurotoxins contained inside the endosomes of the presynaptic terminal must cross the hydrophobic barrier of the endosome membrane. How a large water-soluble protein, such as TeNT and BoNTs, crosses the hydrophobic membrane of the endosome to deliver the catalytic part of the toxin into the cytosol is the least known among the mechanisms of the intoxication of clostridial neurotoxins. In addition to H_C_ (the receptor-binding domain) and H_N_ (translocation domain), the crystal structure of the clostridial neurotoxins also revealed a belt region that wraps between the heavy chain and light chain of clostridial neurotoxins. The acidic interior of the endosome is required for the intoxication to occur. In fact, the agents that interfere with intracellular vesicular acidification inhibit the toxicity of these neurotoxins [141,142]. The N-terminal portion of the heavy chain (H_N_) is highly homologous (60%) among the various clostridial neurotoxins [70], and from the crystal structure, it consists of a pair of unusually long and twisted long α-helices [66,110,143,144]. It has been demonstrated that H_N_ is the minimum channel formation unit, and the belt region is not required [145]. It is unclear where the disulfide bond linked to the LC and HC is reduced. It has been found that recycling endosomes, late endosomes, and lysosomes are not reduced but oxidized and comparable with the conditions in the endoplasmic reticulum, suggesting the a reduction does not happen in the endosome [146]. The examination of the translocation of the LC of BoNT/A and /E through the HC channel showed that a disulfide bridge is required during LC translocation, suggesting the reduction happens in the reducing environment of cytosol rather than in the endosome [145]. Interestingly, without the H_C_, the H_N_ can form the channel at a neutral pH, indicating that the H_C_ alters the pH threshold for membrane insertion [145]. Therefore, in addition to its role in receptor binding, the H_C_ may also play a role in the translocation of toxins. The channel formed by the H_N_ is about 15 Å; therefore, the LC has to undergo a partial unfolding in order to be translocated. The low pH triggered the unfolding process, and the LC from BoNT/A is in a molten globule state at a low pH [147,148,149]. The LC of TeNT also showed pH-dependent plasticity [150]. Collectively, the flexible molten globule structure adopted by the LC at a low pH is required for translocating a large globule protein through the 15 Å channel.

##### Retrograde of Clostridial Neurotoxins to the CNS

**Figure 5:**
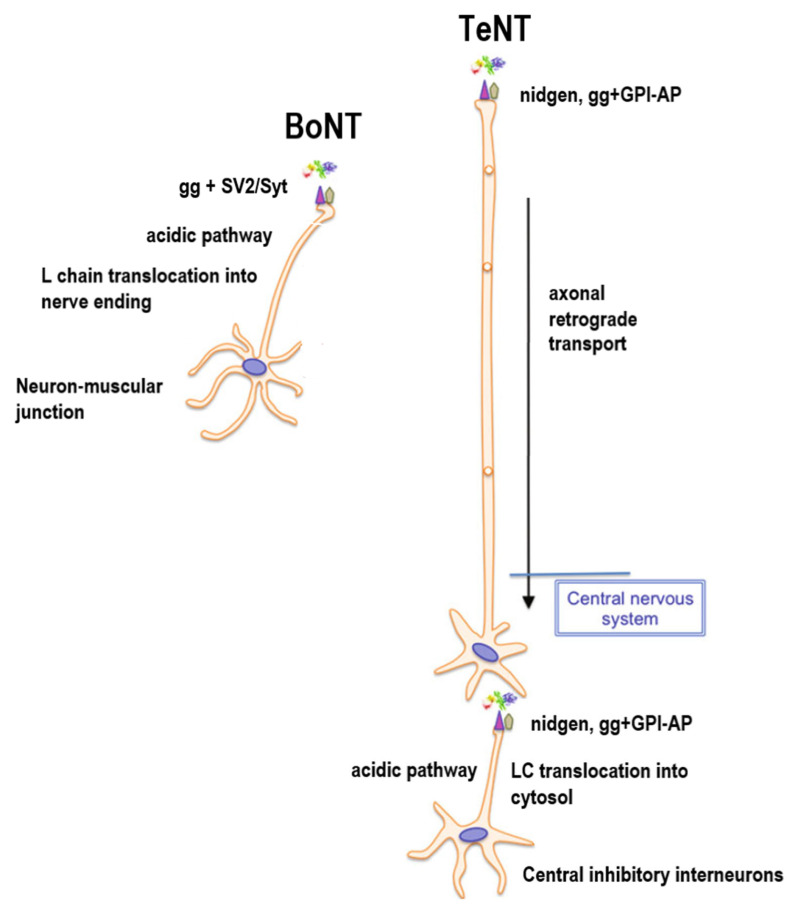
Target neurons of BoNTs and TeNT and their trafficking (modified from Connan and Popoff 2017 [99]). BoNTs target motor neuron endings at the neuromuscular junction, while TeNT enters the motoneuron and sensory neuron endings, moves retrogradely along the axon, is transcytosed, and reenters the central inhibitory neurons.

BoNTs mainly target the motor neuron endings at the neuromuscular junction. TeNT, however, enters the motoneuron, as well as the sensory neuron endings, and moves retrogradely along the axon, is transcytosed, and reenters the central inhibitory neurons (Figure 5) [151]. Like BoNTs, TeNT also uses gangliosides as its receptor, as well as an uncharacterized glycophosphatidylinositol (GPI)-anchored protein as the protein receptor [135]. While SV2A and SV2B have been shown to facilitate TeNT entry into cultured hippocampal/cortical neurons [136], the direct interactions of TeNT with SV2 proteins still need to be confirmed. TeNT entered motor neuron and sensory neuron endings through clathrin-mediated endocytosis [152]. The internalization of TeNT is dependent on a special subset of clathrin adaptors, which direct the toxin to nonacidified endosomal compartments and prevent the translocation of the L chain into the cytoplasm of the motor neuron [153]. The HC of TeNT is believed to drive the retrograde transport of the toxin [154,155]; however, recent studies have also showen that the entire TeNT is required for efficient retrograde transport, suggesting that the regions outside HC also play roles in intracellular trafficking [156,157]. The small GTPases Rab5 and Rab7 have been found to act sequentially in controlling the axonal retrograde transport of TeNT HC in motor neurons [158]. This pathway is shared with p75NTR, TrkB, and BDNF, which is strictly dependent on the activities of both Rab5 and Rab7. The retrograde transport of TeNT is mediated by neutral pH endosomes, similar to the retrograde transport of neurotrophic factors [159]. Understanding the retrograde transport of TeNT is not only critical in understanding the mode of action of TeNT but also provides a great tool to understand the axonal retrograde transport of signaling endosomes [159,160].

BoNTs share a similar structure/function with TeNT. Could BoNT also use certain neuronal cells to enter the central neuronal system in a transcytosis manner? Antonucci et al. first demonstrated that, 3 days following unilateral BoNT/A delivery to the hippocampus, cleaved SNAP25 was detected in the contralateral side [106]. They also demonstrated that the injection of BoNT/A into the optic tectum led to the appearance of BoNT/A-truncated SNAP-25 in synaptic terminals within the retina, and injection of the toxin into rat whisker muscles led to the cleaved SNAP-25 detected in the facial nucleus. Those results suggest that BoNT/A undergo long-distance retrograde transport [106]. The retrograde transport of BoNT/A is microtubule-dependent [161,162,163]. It is further demonstrated that BoNT/E also undergo retrograde axonal transport in primary motor neurons, and BoNT/A, BoNT/E, and TeNT are transported by the same nonacid axonal organelles and remain enzymatically functional in the soma of the motor neurons [151,164]. The uniqueness of BoNTs is that they are not only the most toxic substance to mankind but also an effective medicine for many neuromuscular disorders. The quantities used for the experiments that demonstrated the retrograde transport of BoNT/A and /E are much higher than the therapeutic dosage, and no clinical adverse effects associated with the retrograde transport have been observed for applications of botulinum neurotoxin-based therapeutics.

##### Enzymatic Activity

Once reaching the cytosol of the targeted neurons, the clostridial neurotoxin light chain binds and cleaves the transmembrane SNARE proteins on the membrane of the synaptic vesicle (v-SNARE or vesicle-SNARE) or SNARE proteins on the plasma membrane (t-SNARE or target-SNARE). The cleavage of SNARE proteins prevents SNARE complex formation and, therefore, blocks the vesicle docking to the plasma membrane and the inhibition of the neurotransmitter release from the synaptic cleft [1,8,13,165,166]. The LC of both BoNTs and TeNT share a highly conserved 20-residue-long segment, including a HEXXH zinc-binding domain [1]. Unlike other zinc-metalloproteases, however, the substrates of clostridia neurotoxins are highly specific; they only target SNARE proteins, and different serotypes have either different substrates or different cleavage sites of the same target, except for TeNT sharing the same cleavage site of BoNT/B (Table 2). The LC of BoNT/A, /E, and /C target synaptosomal-associated protein 25 kDa (SNAP-25, a t-SNARE), and BoNT/C also targets syntaxin (another t-SNARE). BoNT/B, /D, /F, /G, /H, /X, and /En target synaptobrevin (also called vesicle-associated membrane protein or VAMP, a v-SNARE). The specificity of LC of clostridial neurotoxins is directed toward unique peptide bonds within the sequence of the respective SNARE substrates (Table 2). As an enzyme, the catalytic efficiency of LC of clostridial neurotoxins is not very high. The average range of k_cat_/K_M_ for BoNT/A LC (the most characterized serotype) is 10^4^–10^6^ s^−1^M^−1^ when using the SNAP-25 fragment > 61 residues or full-length SNAP-25 as the substrate [167] (as a comparison, the turnover number of acetylcholinesterase is 1.5 × 10^8^ s^−1^M^−1^). Yet, it is highly potent to nerve cells, and it has been estimated that as few as 1000 molecules of BoNT are sufficient to inactivate nerve transmission in a muscle [8,168]. Further, the paralysis caused by BoNT can last weeks to months, depending on the serotypes of BoNT. BoNT/A-induced paralysis has the longest duration (BoNT/A-induced paralysis lasts 3–6 months in humans) [169]. The duration of paralysis induced by BoNT/C was shown similar to that of BoNT/A, suggesting BoNT/C may be an alternative for BoNT/A as a therapeutic agent [170,171]. BoNT/B has a shorter duration than BoNT/A of 12–16 weeks in humans [172], while BoNT/E has the shortest duration of 2–7 weeks in humans [173]. The durations of BoNT/D and /F have a similar paralysis duration to that of BoNT/B [174]. The paralysis caused by BoNT is reversible with time, as the clostridial toxin action is blocking the neurotransmitter release rather than killing cells. The question that remains is how an enzyme working intracellularly can induce such a long duration, given the average protein in a eukaryote cell has a typical half-life of ~1.5–48 h [175,176,177,178]. Two factors affect the duration of clostridium neurotoxins: the degradation pathway of LC and the effect of the cleaved substrate on synaptic vesicle docking and exocytosis.

The lifetime of LC has been investigated mostly in vitro due to the high toxicity of BoNTs in vivo. The studies have been mostly focused on BoNT/A and /E, because they display the longest and shortest durations in vivo, respectively. It appears that the BoNT/A LC has a longer lifetime than that of BoNT/E. Tsai et al. demonstrated that the LC of BoNT/E is ubiquitylated and rapidly degraded through the ubiquitin–proteasome system in cells, while the LC of BoNT/A appears to escape this degradation pathway through recruiting deubiquitinases [188,189]. The LC of BoNT/A also showed using dileucine at its C terminus to recruit septins and stabilize the LC in cultured neuronal cells [190]. BoNT/A1, /A2, /A4, and /A5 have shown to remain intracellular enzymatically activite for at least 10 months and BoNT/A3 enzymatically active for up to 5 months in primary rat spinal cord cells [191], supporting the long lifetime of BoNT/A in cells.

Besides the stability of BoNT within cells, the cleavage products may also play roles in the duration of clostridial neurotoxins. BoNT/A and /C showed similar paralysis durations in vivo, while BoNT/E has a much shorter duration. BoNT/A and /C cleave only nine and eight residues from the C-terminal of SNAP-25, respectively, while BoNT/E cleaves off 26 residues from the C-terminal of SNAP-25. The cleaved SNAP-25 by BoNT/A and /C still can bind with VAMP and syntaxin, but the complex is unable to fuse in response to an action potential and Ca^2+^ infusion [192]. The truncated SNAP-25 may remain passively stable in the SNARE complex for a long time and block the exocytosis [8,193]. The SNAP-25 cleavage product by BoNT/E, however, is not able to form a SNARE complex and is not stable inside the nerve cell [192]. Therefore, BoNT/E-cleaved SNAP-25 is not able to block SNARE complex fusion for a long time.

The localizations of the LC in the cytosol are different among different serotypes and may also be responsible for the duration of paralysis caused by different serotypes of BoNT. The BoNT/A L chain was found to be mostly associated with the plasma membranes and colocalized with truncated SNAP-25, whereas the BoNT/B L chain was distributed throughout the cytoplasm. The BoNT/E L chain, however, was located in the cytoplasm but towards the outer periphery in an annular form [194,195]. The differential localization is suggested to be responsible for the variation in the longevity of the L chains of BoNT/A, /B, and /E [194,195].

### 2.3. Molten Globule-Type Structures Are Critical for the Action of Clostridial Neurotoxins

The crystal structures of clostridial toxins have been resolved. However, the crystal structure alone cannot explain all the functions of BoNTs and TeNT, and as a result, there no effective inhibitors have been identified against Clostridium neurotoxins, especially their endopeptidase activity [165,166]. This reflects that the crystal structure may not completely represent the functional active structure of clostridial neurotoxins. Clostridial neurotoxins are produced as a single-chain polypeptide with a nicking site. Toxins need to be nicked into a dichain form, which is still linked through the disulfide bond to be activated [8]. Endopeptidase activity is activated only after the interchain disulfide bond is reduced [8]. However, the reduced toxins do not show toxicity, and the interchain disulfide bond is required for translocation [196,197,198,199]. During membrane translocation and enzymatic action, the structures of toxins are adopted as functional structures that might be different from the crystal structure. The flexible structures of clostridial neurotoxins are needed for both translocating the enzymatic domain through the endosomal membrane and their enzymatic activity [8,147,150,165,166]. During the translocation step, to transport the toxin through the membrane, a 50-kDa light chain is adopted as a molten globule-type structure in the low pH environment of the endosome [147]. The HC of the toxin behaves as a chaperon helping the LC pass through the narrow membrane channel to reach the cytosol [145,150].

**Figure 6:**
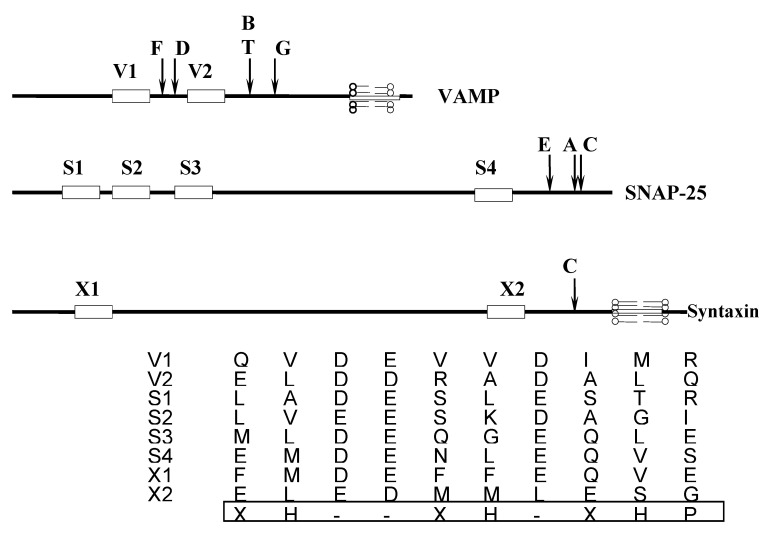
Positions of the SNARE motif in three substrates responsible for the target specificity of clostridial neurotoxins [200]. The motif consists of nine residues that are common to all three substrates: hydrophobic residue (H), Asp or Glu residue (-), polar residue (P), and any residue (X).

One of the uniqueness of clostridial neurotoxins is their highly selective substrates. The LCs of clostridial neurotoxins are highly selective for the three SNARE proteins, which are cleaved at different peptide bonds (Table 2). This exquisite selectivity is believed to be due to the presence of a highly conserved common motif, called the SNARE motif, in the amino acid sequence of the target [200]. Depending on the protein, the motif is termed V1 and V2 in VAMP, S1–S4 in SNAP-25, and X1 and X2 in syntaxin (Figure 6). These motifs are predicted to adopt a helical conformation with three negative charges contiguous to a face formed by hydrophobic residues (Figure 6) [200]. The crystal structures of the substrate LC were resolved for BoNT/A-SNAP-25 and BoNT/F-VAMP [201,202]. Those results, along with the biochemical evidence, point to the multiple interactions of the LCs with their substrates, including the active site and the exosites located outside the active site [201,203,204]. However, crystal structures cannot adequately explain the experimental results. For example, α-exosite on the BoNT/A L chain is shown to interact with residues in the vicinity of the S4 SNARE motif (but not with the S4 residues) of SNAP-25 [201]. Yet, it is notable that S4, as well as other SNARE motifs, are able to enhance the endopeptidase activity of the BoNT/A L chain [205,206]. The cocrystal structure also clearly shows that the C-terminal residues (201–204) of the SNAP-25 create a unique three-stranded antiparallel β-sheet in the *β*-exosite region, which forms a critical interface between SNAP-25 and the “250 loop” of the BoNT/A L chain [201]. However, a shorter synthetic substrate containing the BoNT/A cleavage site, but with none of the critical residues except Lys-201, is readily and specifically cleaved by the BoNT/A L chain [207]. SNAP-25 is reported as an unstructured protein [208,209]. The cocrystal of the BoNT/A L chain with truncated SNAP-25 also shows largely unstructured SNAP-25 [201]. The specificity of the enzyme and substrate may rely on a dynamic state of interaction that has not been observed experimentally in the crystal structure. Many enzymatic reactions occur on time scales of micro- to milliseconds; it is anticipated that the conformational dynamics of the enzyme at these time scales might be linked to its catalytic action [210]. The flexible structures such as molten globules may hold the key to such molecular events at a time scale that cannot be ordinarily evaluated from the crystal structures [211].

**Figure 7:**
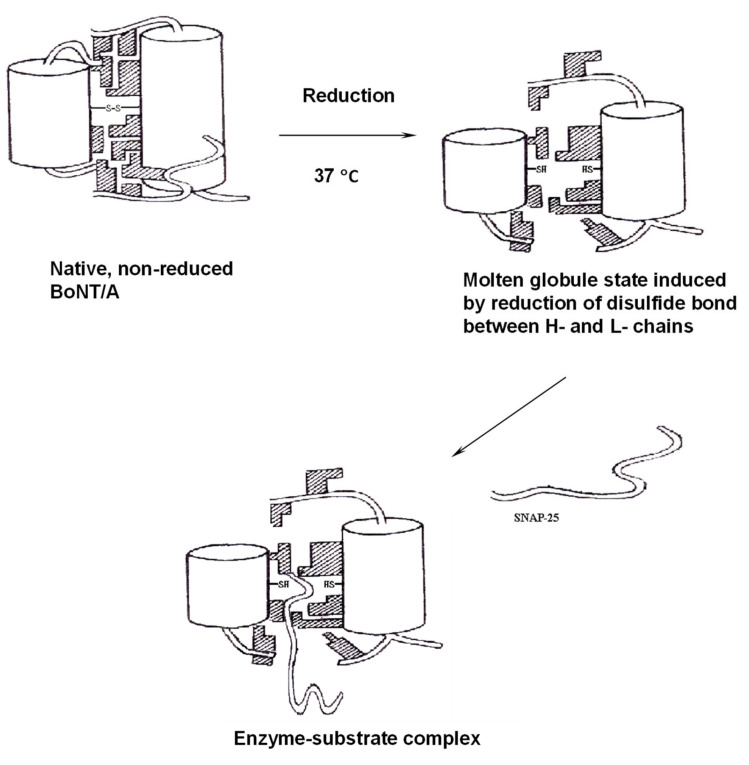
Schematic model of the reduction-induced molten globule state of BoNT/A facilitating the binding of SNAP-25 [212].

Clostridial neurotoxins are initially synthesized as a single polypeptide chain and then are nicked by either endogenous or exogenous proteases to form the dichain linked through a disulfide bond. The dichain form of toxins is believed to be biologically active [8]. In addition to proteolytic activation, the disulfide bond is also crucial for the endopeptidase activity of BoNT and TeNT. The overall structural changes of toxins upon nicking and reduction are responsible for the activation of endopeptidase activity. It has been reported that the activation mechanism of BoNT/A by reduction of the interchain disulfide bond involves a molten globule state under physiological temperature conditions, while nonreduced BoNT/A does not have this state [212]. The reduction of the interchain disulfide bond also includes a more flexible structure with higher enzymatic activity for BoNT/E [213]. The reduction is believed to happen in the cytosol, with a high reducing potential (the interchain disulfide bond is required for the translocation). The reduction-induced molten globule-type conformation of BoNT/A and /E plays a critical role in its intracellular toxification process, and yet, it is difficult to capture it with a crystal structure in vivo. This molten globule conformation may facilitate the binding of unstructured SNAP-25, thus facilitating its cleavage by the endopeptidase activity of BoNT (Figure 7) [212].

**Figure 8:**
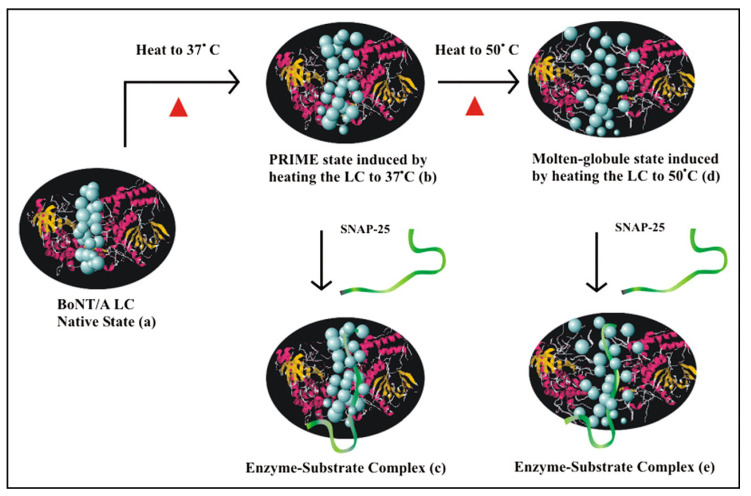
Schematic model representing the PRIME state and the molten globule state in BoNT/A LC facilitating the binding of substrate SNAP-25. The blue balls represent the nonpolar side chains, which are tightly packed in the native conformation. (**a**) Heating to 37 °C significantly alters the polypeptide folding, and the protein core becomes slightly loosened compared to the native state (**b**) and facilitates the binding of SNAP-25 to form the enzyme–substrate complex (**c**). BoNT/A LC exhibits the optimum activity in this PRIME state. The further heating of the BoNT/A LC leads to the formation of a molten globule intermediate, which is represented by a loose packing of the side chains in the protein core and partial unfolding of loops with a slightly more expanded structure than PRIME (**d**). While less optimal, this state of the BoNT/A LC also binds to SNAP-25, forming the enzyme–substrate complex, (**e**) and displays less enzymatic activity than the optimal PRIME state [209].

LC alone is thought to be presented inside the nerve cells during BoNT/TeNT poisoning nerves. The conformation of active LC endopeptidase is needed to correlate with its specific SNARE targets. There is strong evidence for the molten globule conformation present in the BoNT/A L chain, whose conformation and functional profile is different from that of BoNT/A. The molten globule conformation of the BoNT/A LC is observed at 50 °C (vs. a reduced BoNT/A at 37 °C). While this molten globule state may not be relevant under normal physiological conditions, more intriguingly, BoNT/A LC is part of the existence of another intermediate state at 37 °C that precedes the molten globule state and that is more expanded and dynamically flexible but distinctly different from the molten globule state. This intermediate exhibits optimum enzymatic activity and is referred to as the Pre-Imminent Molten-globule Enzyme (PRIME) conformational state [214]. More recently, using small-angle X-ray scattering combined with the fluorescence lifetime and a molecular dynamics simulation, it has been confirmed that the intramolecular mobility of the PRIME state of BoNT/A LC is significantly higher than that of the native state, which can be attributed to the increased dynamics and expansion of the protein core [210,215]. This extended solution structure facilitates the maximum specific binding of SNAP-25, leading to its cleavage. The molten globule conformation, on the other hand, has a considerably intact secondary structure, loose packing of side chains in the protein core, and partial unfolding of the loops [214]. This structure also binds to SNAP-25, although to a lesser degree than the PRIME state, thus showing only about 61% of the optimum enzymatic activity [214]. Figure 8 represents a schematic model of the temperature-induced PRIME conformational and molten globule states of the BoNT/A LC, showing how these states in the BoNT/A light chain may facilitate the binding and cleavage of SNAP-25 [214].

The PRIME confirmation state is also observed in the BoNT/B LC and appears to be responsible for its optimal enzymatic activity at 37 °C [216]. The BoNT/E LC, however, adopts an active conformation that differs from the PRIME conformation of LCA and LCB [216]. The BoNT/E LC is in a molten globule state at 47 °C, which is also optically active enzymatically with the full-length substrate, similar to reduced BoNT/E toxin [213,216]. Differential features of the structural and functional characteristics of these active molten globule-related structures among the three BoNT may be relevant to their specificity with the substrates and their survival intracellularly.

The length of the LC of BoNT is critical to maintaining the active structure. The crystal structure of the recombinant BoNT/A LC truncated 24–30 amino acids from its C-terminus (the full length of the BoNT/A LC is 1-448), mainly due to the lower solubility of the full length of the BoNT/A LC [201,217]. The enzymatic activity for the truncated LC of BoNT/A, however, is markedly lower than the full-length LC [218]. The detailed structural analysis in solution revealed that the differences of the flexibility between the truncated and full-length LC and the 24-residue at the C-terminus play a critical role in introducing a flexible structure that is essential for its endopeptidase activity [218].

The NAPs of BoNT are mainly believed to play a protection role and help the transcytosis of BoNT. However, in vitro enzymatic studies showed that the complex form of the toxin without a reduction of the disulfide bond exhibits higher endopeptidase activity similar to the reduced toxin [219,220]. While the physiological relevance of this observation remains to be answered, the enhanced enzymatic activity may be due to the interaction of the NAPs with BoNT and inducing structural changes to the active form similar to the reduced toxin [219,220]. Indeed, Hn-33 from BoNT/A alone not only enhances the endopeptidases activity of BoNT/A but also enhances the enzymatic activity of BoNT/E [221]. The structures of the L complexes of BoNT/A, /B, and /D have been studied using a negative staining electron microscope and show that hemagglutinin proteins are on the opposite side of the BoNT-NTNHA interface [78,222,223]. The solution binding study, however, showed that Hn-33 from BoNT type A can directly bind to BoNT/A, as well as to BoNT/E [94,221]. The interaction of the toxin and Hn-33 alone could change the structure of the toxin to a more flexible state than the substrate can access [221].

The flexible molten globule conformation of BoNT observed at a certain physiological temperature may facilitate the binding of unstructured SNAP-25, thus facilitating its cleavage by endopeptidase activity, as well as substrate specificity. Although the zinc-binding motif HExxH in the active site is identical for all clostridial neurotoxins, different types of BoNT and TeNT recognize either a different substrate or at least a different cleavage site, except for TeNT and BoNT/B, both of which recognize synaptobrevin and the same cleavage site. The different forms of the flexible structures/molten globule states of different clostridial neurotoxins, along with the SNARE motifs of their respective SNARE substrates, may be vital in recognition specificity and enzymatic activity. This mechanism provides the reconciliation of the apparent inaccessibility of the BoNT/A endopeptidase active site, as suggested by the X-ray crystallographic structure [66,110,224]. The flexible structures of clostridial neurotoxins in the solution highlight the challenges facing the design of effective inhibitors against this group of toxins. Even though the crystal structures were resolved for BoNTs and TeNT more than a decade ago, still, no effective inhibitors were identified for their endopeptidase activity, partially due to the disconnection of the crystal structure and the active structure [165,166,210,215,225].

## 3. Flexible Structures Are Utilized as Active Structures beyond Clostridial Neurotoxins

The molten globule-type structure is characterized by the existence of native-like secondary structures and the native-like folding pattern with a loosely packed nonpolar core without a rigid tertiary structure [212,226,227]. The molten globule state is believed to be an intermediate state during protein folding and as a translocation competent state in the transport of a precursor protein across biological membranes [228]. It has been found that molten globule proteins can adopt the native topology and bind targets tightly and specifically and may play roles in molecular recognition in folding and/or binding reactions [229]. A protein can adopt different molten globule states depending on the biological process. For clostridial neurotoxins, the LC adopted a molten globule state during the translocation step [147], and the enzymatic activity also required a molten globule state induced by a reduction of the interchain disulfide bond [212]. While no experimental data on the structure changes during the protease activation step, the activation must also be associated with the structural changes. The formation of a molten globule state under physiological conditions may be closely related to their respective biological functions. In addition to clostridial neurotoxins, many protein toxins, including Gram-positive bacterial toxins, also adopt flexible structures for their biological functions.

A molten globule-type structure is one of the mechanism protein toxins used for membrane insertion, including Gram-positive bacterial protein toxins. Diphtheria toxin (DT), a 58-kDa exotoxin, produced by the Gram-positive *Corynebacterium diphtheriae*, is a A-B type toxin. Like clostridial neurotoxins, DT is synthesized as a single-chain peptide and needs proteolysis activation to form a dichain (21-kDa fragment A and 37-kDa fragment B) linked through an interchain disulfide bond [9]. The C-terminus of fragment B is responsible for receptor binding (R domain), and the N-terminus of fragment B is responsible for translocation (T domain), while fragment A is the catalytic domain to inactivate the eukaryotic elongation factor 2 (EF2) through ADP-ribosylation and inhibit protein synthesis. At a low pH, the T domain interacts with membrane molten-globule-like proteins that partially unfold and increase their hydrophobicity, permitting a deep insertion of the T domain into the membrane [230]. The low pH environment within the endosome triggers the partial unfolding of fragment A, and this molten globule-type structure is believed to facilitate the translocation [231,232].

Anthrax toxin, produced by the Gram-positive bacterium *Bacillus anthracis*, is an ensemble of three large, multidomain proteins. They are secreted from *Bacillus anthracis* as monomers and self-assemble on receptor-bearing cells into a series of toxic, hetero-oligomeric complexes [233]. Anthrax toxin is composed of two enzymic intracellular effectors: the Lethal Factor (LF, 90 kDa), a Zn^2+^-dependent protease, and the Edema Factor (EF, 89 kDa), a Ca^2+^- and calmodulin-dependent adenylyl cyclase, and a receptor-binding and pore-forming protein, Protective Antigen (PA, 83 kDa), which transports EF and LF to the cytosolic compartment of mammalian cells [233]. Anthrax toxin needs proteolytic activation to cleave the PA protein into N-terminal 20 kDa (PA20) and C-terminal 63 kDa (PA63). PA63 alone can mediate the biological effects of LF and EF, which can lead to the death of the host [233,234]. PA63 has also been explored as a vehicle to deliver therapeutical proteins into cancer cells [235]. PA63 is self-associated and forms a heptameric prepore that binds to EF and LF and the complex of PA7/EF/LF, which are endocytosed. The low pH within the endosome triggered the partial unfolding of EF/LF into molten globule-type structures. This molten globule state of EF/LF is critical for the translocation of EF/LF into the cytosol [236].

Another class of toxins that interacts with the cell membrane are pore-forming toxins, which represent more than one-third of all bacterial toxins. Many Gram-positive bacterial toxins belong to pore-forming toxins, including *Clostridium perfringens* perfringolysin O and Staphylococcal α-toxin. Others have intermediate functions between straightforward pore-forming toxins and A–B-type toxins, such as *Streptococcus pyogenes*, which uses streptolysin O, a pore-forming toxin belonging to the cholesterol-dependent cytolysin family, to translocate the NAD:glycohydrolase enzyme directly into the target eukaryotic cytosol components [237]; intracellular pathogen *Listeria monocytogenes* produces listeriolysin, another member of the cholesterol-dependent cytolysin family. Unlike other cholesterol-dependent cytolysins that induce pores at both low and neutral pH conditions, listeriolysin is only activated at a low pH [238]. Partially unfolded structures play critical roles in pore-forming toxins [230]. Perfringolysin O, a member of cholesterol-dependent cytolysin, is a water-soluble protein and, upon binding to the receptor on the lipid membrane, forms an oligomerized prepore formation. The pore formation requires the unfolding of α-helices on the D3 domain into two β-hairpins and insertion of the resulting β-barrel into the lipid bilayer [239]. Other cholesterol-dependent cytolysins, such as pneumolysin from Gram-positive *Streptococcus pneumoniae*, streptolysin O, and listeriolysin, also share a similar mechanism for pore formation, and structural changes are required for pore formation upon binding to the cholesterol and the lipid membrane [238,240,241]. Like cholesterol-dependent cytolysins, *Staphylococcus* α-toxin is secreted as a soluble monomeric protein and interacts with the lipid bilayer in a similar way to form a pore. Hydrophilic monomers bind to membrane lipids and, possibly, other specific receptor(s), oligomerize, and unfold the C-terminal half of the alpha-toxin into a molten globule-like state, which is required for channel formation, though α-toxin and related toxins heptamerize and only insert one amphipathic transmembrane hairpin as a monomer into the β-barrel, yielding a small pore [230,242].

Molten globule conformation permits the protein molecule to adapt to a wide variety of external conditions and has been implicated in physiological processes such as membrane insertion and pore formation [230,243] and protein receptor recognition [244], as well as enzymatic activity [245]. Proteolytic activation and disulfide reduction are not unique to the activation of clostridial neurotoxins. Many bacterial toxins and viral proteins use a similar activation mechanism. The spike protein from SARS-CoV-2 requires proteolytic activation to change the conformation of its receptor-binding domain to bind to its receptor, human angiotensin-converting enzyme 2 [246]. While the conformation of the active receptor-binding domain of the spike protein is unknown, it is clear that the changed conformation is adopted to its receptor to enable binding to the receptor [246]. The disulfide bond reduction inducing structural changes is another activation mechanism. In addition to clostridial neurotoxins, disulfide bond reduction is also required for the activation of catalytic domain DT [9]. While molten globule-type structures provide flexibility for different functions, native-like folding may represent a more stabilized structure that permits retention of the important features of its overall architecture. Therefore, many bacterial toxins and virus proteins are synthesized as a nonactive form that needs to be activated when reaching their targets to adapt to their respective activated structures. This adaption may come from natural evolution and selection. The ancient bacterial toxins, like clostridial neurotoxins, prove to be a great example for understanding natural selection and evolution [1].

For decades, there has been increasing conviction that the biological functioning of proteins can be interpreted only in terms of their three-dimensional (3D) structures, and ordered native structures generally obtained from X-ray crystallography have been accepted as the standard structure [8,247]. However, it is very difficult, if not possible, for X-ray crystallography to capture the molten globule-type intermediates and semiflexible structures [247]. This poses a great challenge for design antidotes against bacterial toxins and viruses, including clostridial neurotoxins. While NMR may provide an alternative to capture the molten globule state [227,248], it needs a high protein concentration, and the structure determined from NMR may not be correlated to that at the physiological conditions. For example, BoNT/A adopted a different conformation at the physiological concentration compared to a high concentration [220]. The role of molten globule-type flexible structures is not only important in understanding the fundamental protein folding mechanism but also critical in protein functions (including Gram-positive bacterial toxins). The multidimensional spectroscopic approaches combined with molecular dynamics simulations provide important tools in understanding the molten globule-type flexible structures of proteins [210,248,249]. With the increased computational capacity, molecular dynamics simulations play a more and more important role in understanding molten globule structures [210,250]. Using multidimensional approaches by a combination of high-resolution structural biology tools such as X-ray crystallography and NMR, solution spectroscopic methods and computational approaches such as molecular dynamics simulations will be important in designing effective antidotes against bacterial toxins [215,251,252,253].

## 4. Concluding Remarks

Gram-positive bacteria are ancient organisms. They belong to the earliest lifeform on the Earth billions of years ago. Bacterial toxins, just like high-precision molecular machines, fulfill their functions through different domains and folding. Clostridial neurotoxins provide a classic example in this regard. The diversity of botulinum neurotoxins may be the result of horizontal gene transfer through billions of years of evolution [1]. The molecular machine of clostridial neurotoxins is perfectly fitted into their functions through receptor recognition and binding to the translocation of the virulent factor (LC) into the targeted cells to selectively cleave the target substrates. During each step, the folding of clostridium neurotoxins and their domains is adapted to different flexible structures to fit their functions. The activation and flexible structures of toxins are common among different Gram-positive bacterial toxins. Those indicate that they all come from common ancient ancestors. Indeed, protein folding is critical for biological functions and evolves much more slowly compared to genetic codes, providing a vital tool to understanding natural selection and evolution [254]. The importance of molten globule-type flexible structures for bacterial toxins like clostridial neurotoxins also highlights the need for how to probe those flexible structures. X-ray crystallography and other high-resolution techniques may not be enough, and multidimensional approaches targeting the solution phase are required to understand this type of highly dynamic flexible structure and better understand the modes of action of those toxins. Understanding those highly dynamic structures that are directly related to their function is vital for designing effective antidotes for bacterial toxins and the better utilizing of those toxins as potential medical treatments for different diseases.

## Figures and Tables

**Table 1:** Protein receptors for clostridial neurotoxins.

Toxin Type	Receptors	References
BoNT/A	SV2A, SV2B, SV2C	[123,124]
BoNT/B	SYT-I, SYT-II	[125,126,127]
BoNT/C	Not identified, unknown SV structures	[118,120,121]
BoNT/D	SV2A, SV2B, SV2C	[128]
BoNT/E	SV2A, SV2B	[118,129]
BoNT/F	SV2A, SV2B, SV2C	[130]
BoNT/G	SYT-I, SYT-II	[126,127,131,132,133]
BoNT/H (BoNT/FA)	SV2A, SV2B, SV2C	[134]
BoNT/X	not identified	
TeNT	GPI-anchored protein, nidogen SV2A/B?	[135,136]

**Table 2:** Clostridial neurotoxin targets and peptide bond specificities.

Toxin Type	Target	Cleavage Site	References
BoNT/A	SNAP-25	Ala-Ser-Gln^197^–Phe^198^-Glu-Thr	[179]
BoNT/B; TeNT	VAMP	Ala-Ser-Gln^78^–Phe^79^-Glu-Thr	[180]
BoNT/C	SNAP-25	Ala-Ser-Gln-Phe^198^–Glu^199^-Thr	[169]
	Syntaxin	Thr-Lys-Lys^252^–Ala^253^-Val-Lys	[181]
BoNT/D	VAMP	Asp-Gln-Lys^61^–Leu^62^-Ser-Glu	[182,183]
BoNT/E	SNAP-25	Ile-Asp-Arg^180^–Ile^181^-Met-Glu	[184,185]
BoNT/F	VAMP	Arg-Asp-Gln^60^–Lys^61^-Leu-Ser	[108]
BoNT/G	VAMP	Thr-Ser-Ala^83^–Ala^84^-Lys-Leu	[186,187]
BoNT/H (FA)	VAMP	Lys-Val-Ile^56^–Glu^57^-Arg-Asp	[27,30]

## Data Availability

Not applicable.
